# Absence of BRINP1 in mice causes increase of hippocampal neurogenesis and behavioral alterations relevant to human psychiatric disorders

**DOI:** 10.1186/1756-6606-7-12

**Published:** 2014-02-14

**Authors:** Miwako Kobayashi, Toshiyuki Nakatani, Toshiaki Koda, Ken-ichi Matsumoto, Ryosuke Ozaki, Natsuki Mochida, Keizo Takao, Tsuyoshi Miyakawa, Ichiro Matsuoka

**Affiliations:** 1 Laboratory of Physiological Chemistry, College of Pharmaceutical Sciences, Matsuyama University, 4-2 Bunkyo-cho, Matsuyama, Ehime 790-8578, Japan; 2 Laboratory of Neuroscience, Graduate School of Pharmaceutical Sciences, Hokkaido University, Nishi 6, Kita 12, Kita-ku, Sapporo 060-0812, Japan; 3 Laboratory of Embryonic and Genetic Engineering, Graduate School of Life Science, Hokkaido University, Kita 21, Nishi 11, Kita-ku, Sapporo 001-0021, Japan; 4 Department of Biosignaling and Radioisotope Experiment, Interdisciplinary Center for Science Research, Organization for Research, Shimane University, 89-1 Enya-cho, Izumo 693-8501, Japan; 5 Section of Behavior Patterns, Center for Genetic Analysis of Behavior, National Institute for Physiological Sciences, 38 Nishigo-naka Myodaiji, Okazaki 444-8585, Japan; 6 Japan Science and Technology Agency, CREST, Kawaguchi 332-0012, Japan; 7 Division of Systems Medical Science, Institute for Comprehensive Medical Science, Fujita Health University, 1-98 Dengakugakubo, Kutsukake-cho, Toyoake 470-1192, Japan

**Keywords:** Hippocampal neurogenesis, Interneuron, Behavior, Psychiatric disorder

## Abstract

**Background:**

We have previously identified BRINP (BMP/RA-inducible neural-specific protein-1, 2, 3) family genes that possess the ability to suppress cell cycle progression in neural stem cells. Of the three family members, BRINP1 is the most highly expressed in various brain regions, including the hippocampus, in adult mice and its expression in dentate gyrus (DG) is markedly induced by neural activity. In the present study, we generated BRINP1-deficient (KO) mice to clarify the physiological functions of BRINP1 in the nervous system.

**Results:**

Neurogenesis in the subgranular zone of dentate gyrus was increased in BRINP1-KO mice creating a more immature neuronal population in granule cell layer. The number of parvalbumin expressing interneuron in hippocampal CA1 subregion was also increased in BRINP1-KO mice. Furthermore, BRINP1-KO mice showed abnormal behaviors with increase in locomotor activity, reduced anxiety-like behavior, poor social interaction, and slight impairment of working memory, all of which resemble symptoms of human psychiatric disorders such as schizophrenia and attention–deficit/hyperactivity disorder (ADHD).

**Conclusions:**

Absence of BRINP1 causes deregulation of neurogenesis and impairments of neuronal differentiation in adult hippocampal circuitry. Abnormal behaviors comparable to those of human psychiatric disorders such as hyperactivity and poor social behavior were observed in BRINP1-KO mice. These abnormal behaviors could be caused by alteration of hippocampal circuitry as a consequence of the lack of BRINP1.

## Background

Elucidation of the molecular and pathological mechanisms in the onset of human psychiatric disorders such as schizophrenia remains a great challenge in molecular neuroscience, partly because of the complexity of the diseases with both numerous genetic risk factors and various environmental factors. The lack of quantifiable biological indicators has also hindered objective analyses in human subjects. Therefore, the development of animal models with homogeneous backgrounds should help in clarifying the genetic and molecular contributions to the onset of psychiatric disorders.

Since endophenotype is a concept that lies between genetics and disease, identification and analysis of the endophenotype should contribute towards an understanding of the complicated mechanisms of the development of psychiatric disorders and so to finding a good predictor of the onset of the diseases. Therefore, searches for common phenotypes including structural and behavioral alterations within a group of mutant mice can help us establish the endophenotypes related to particular human psychiatric disorders [1,2]. In this regard, recent studies using a comprehensive behavioral test battery revealed that mutant mice of both susceptible and non-susceptible genes of human psychiatric disorders showed interesting abnormal behaviors relevant to the symptoms of psychiatric disorders [3].

*BRINP1* (*BMP/RA-induced neural specific protein-1*) was identified as a gene which is induced during differentiation of peripheral neurons [4]. With subsequently identified members, BRINP2 and BRINP3, BRINP1 forms a novel protein family (BRINP family). Although the primary structures of these three BRINPs show almost no similarity to any known proteins, they are evolutionally conserved at extremely high levels in vertebrates from fish to mammals [4]. In the mouse nervous system, mRNA of the three BRINPs become detectable from E9.5 and continues to express in adulthood, each with a distinctive pattern. Of these BRINP family genes, BRINP1 is most highly expressed in various brain regions, such as hippocampus, cortex, olfactory bulbs, striatum and cerebellum [4]. We have previously shown that over-expressed BRINPs suppress cell cycle progression in non-neural cells [4] and neural stem cells [5]. Moreover, the induction of BRINP1-mRNA in hippocampus by glutamatergic stimulation suggested that BRINP1 possesses a plasticity-related physiological function in the hippocampus [6].

The hippocampus plays key roles in spatial navigation and memory formation, and its functional impairment causes various neurological disorders such as Alzheimer’s disease, epilepsy and schizophrenia. The subgranular zone (SGZ) of the dentate gyrus (DG) in hippocampus is one of two restricted regions where neurogenesis regularly occurs in normal adult mammalian brain [7,8]. It has been shown that newborn neuronal precursor cells in SGZ differentiate into dentate granule cells, migrate into inner granule cell layer and are integrated into neuronal circuitry [9,10]. These adult-born granule neurons are thought to play important roles in memory formation [11] such as mediating spatial information processing [12]. Numerous studies showed that up- or down-regulation of adult neurogenesis can affect hippocampus-dependent behaviors [13].

In the present study, to elucidate the physiological functions of BRINP1 in the adult brain we generated BRINP1-KO mice and analyzed their phenotypes on various aspects such as neurogenesis, neuronal differentiation and behavioral alterations with specific attention to hippocampal functions. Consequently, we found that the neurogenesis in SGZ was significantly increased, creating a more immature neuronal population in BRINP1-KO mice. The number of parvalbumin expressing interneurons was also increased in hippocampal CA1 subregion in BRINP1-KO mice. Furthermore, we found abnormal behaviors of BRINP1-KO mice which resemble behaviors associated with certain human psychiatric conditions. From these results, we discuss the possible molecular functions of BRINP1 to regulate neurogenesis, neuronal differentiation and survival in hippocampal circuitry.

## Results

### Generation of BRINP1-KO mice

**Figure 1:**
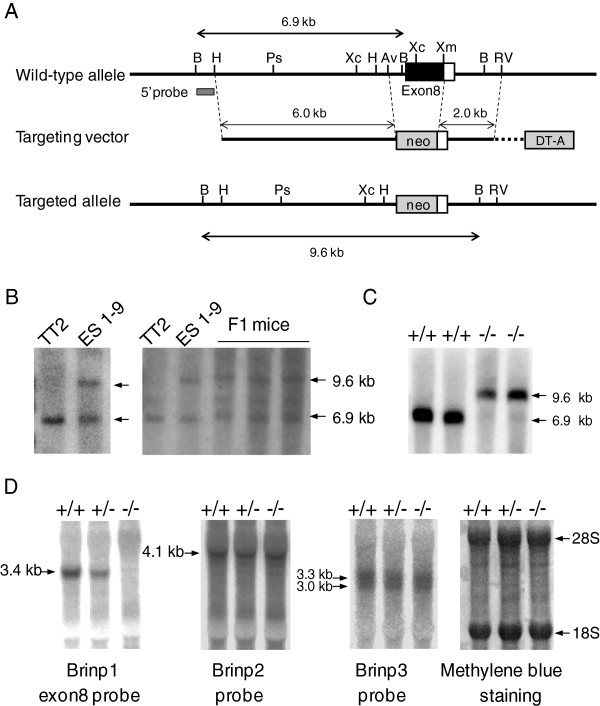
**Targeted disruption of BRINP1 gene in ES cell and mouse. (A)** Construct of the targeting vector for homologous recombination to yield BRINP1-KO mice. The coding region of *Brinp1* exon8 (filled box) was disrupted by PGK-neomycin resistance cassette. The probe used for Southern blot analysis is shown together with predicted sizes of hybridizing fragments. Sites of restriction enzymes: Av, AvrII; B, BamHI; H, HindIII; Ps, PstI; RV, EcoRV; Xc, XcmI; Xm, XmaI. **(B)** Southern blot analysis of BamHI-digested genomic DNA extracted from control (TT2) and positive clone (1–9) of ES cells, and F1 mice produced by crossing chimera mice with C57BL/6J mice. **(C)** Southern blot analysis of genomic DNA extracted from wild-type (+/+) and BRINP1-KO (−/−) mice. **(D)** mRNA expression of BRINP family genes in BRINP1-KO mice. Total RNA extracted from adult brain of wild-type (+/+), BRINP1 heterozygous (+/−), and BRINP1-KO (−/−) mice were hybridized with each of *Brinp1*, *Brinp2* and *Brinp3* antisense probes. Uniform transfer of RNA was confirmed by methylene blue staining.

**Figure 2:**
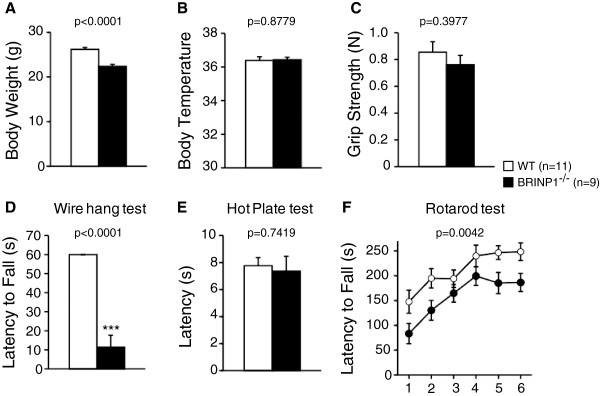
**General health and neurological screening of BRINP1-KO mice. (A)** Body weight of BRINP1-KO mice was reduced to about 85% of wild-type mice. **(B)** Body temperature was measured at rectum. **(C)** Grip strength of forelimb in Newton. **(D)** Latency to fall (s) showed in wire hang test. **(E)** Hot plate test at 55°C. **(F)** Rotarod test. (wild-type mice, n = 11; BRINP1-KO mice, n = 9) Error bars indicate SEM.

BRINP1-KO mice were designed to replace *Brinp1* exon8 with neomycin resistant gene (Figure 1A). Homologous recombination of genomic DNA in embryonic stem (ES) cells and F1 mice was confirmed by Southern blot analysis that produced 6.9 kb and 9.6 kb BamHI fragments hybridized with 5’ probe (Figure 1B-C). Northern hybridization with *Brinp1* exon8-cRNA probe showed that BRINP1-mRNA was absent in the adult brain of BRINP1-KO mice (Figure 1D). Loss of BRINP1 expression did not alter mRNA levels of BRINP2 or BRINP3, the other members of BRINP family genes, suggesting that there is no compensation of mRNA expression among BRINP family genes in BRINP1-KO mice. BRINP1 homozygous KO mice showed a normal appearance at birth and had normal skeleton. The body weight of BRINP1-KO mice (22.38 ± 0.45 g) was about 85% of wild-type (WT) littermates (26.22 ± 0.38 g) at adult stage (Figure 2A). However, there were no significant differences in the weights of either brain or hippocampus between BRINP1-KO and wild-type mice (i.e. brain weight, WT; 774.4 ± 71.3 mg (n = 5), BRINP1-KO; 697.4 ± 96.4 mg (n = 11), hippocampal weight per mouse, WT; 24.6 ± 2.3 mg (n = 10), BRINP1-KO; 25.8 ± 1.8 mg (n = 12)).

### Behavioral alterations in BRINP1-KO mice

To examine the effects of disruption of BRINP1 gene on mouse behavior, we performed a comprehensive behavioral test battery [14] on BRINP1-KO mice. First we examined general health and conducted neurological tests. As mentioned above, body weight was substantially reduced in BRINP1-KO mice compared to that of wild-type littermates. The higher locomotor activity of BRINP1-KO mice in home cage may explain the decrease of body-weight (see Additional file 1: Figure S1A). Body temperature (Figure 2B) and sensory abilities of BRINP1-KO mice appeared to be normal (Figure 2E). BRINP1-KO mice showed normal levels of neuromuscular strength in grip strength test (Figure 2C). On the other hand, BRINP1-KO mice showed a remarkably short latency compared to wild-type mice in the wire hang test (Figure 2D). BRINP1-KO mice also showed shorter latency than wild-type mice in the fall from the rotating drum in Rotarod test (Figure 2F). Since BRINP1-KO mice improved their performance during the repeating trials of Rotarod test in a similar manner to wild-type mice, it seems that their ability to learn motor skills did not decline. These results suggest that either motor coordination was impaired in BRINP1-KO mice or they are mentally less patient than wild-type mice.

**Figure 3:**
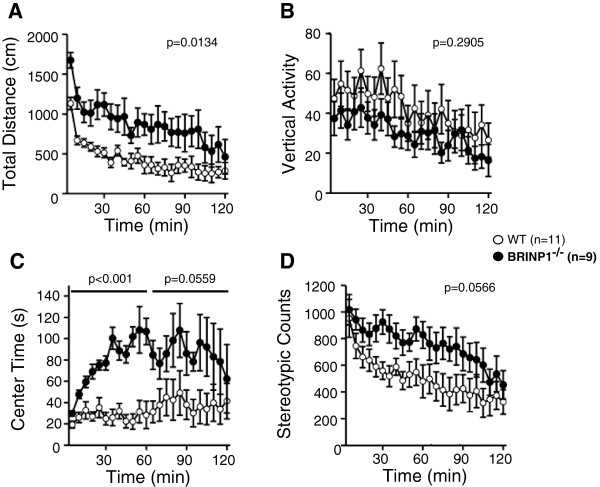
**Exploratory activity in BRINP1-KO mice in open field test. (A)** Total distance traveled. **(B)** Counts of vertical activity. **(C)** Time spent in the center of the compartment. **(D)** Counts of stereotypic behavior. (wild-type mice, n = 11; BRINP1-KO mice, n = 9) Error bars indicate SEM.

Exploratory activity was assessed with the open field tests. Throughout the 120 min observation period BRINP1-KO mice showed more than 1.5 times higher locomotor/exploratory activity than wild-type mice (Figure 3A). Time spent in the center area of the test apparatus of BRNP1-KO mice was markedly longer than that of wild-type mice and the difference was highly significant during the first 60 min of the test (Figure 3C). This result suggests that BRINP1-KO mice are less fearful in the open field. Although there was no significant difference in vertical activity and stereotypic behavior between BRINP1-KO mice and wild-type mice, BRINP1-KO had a tendency to show less vertical activity and more stereotypic behavior compared to wild-type mice. (Figure 3B and 3D). These results from the open field test indicate that the increased locomotor activity in BRINP1-KO mice is accompanied by reduced anxiety.

**Figure 4:**
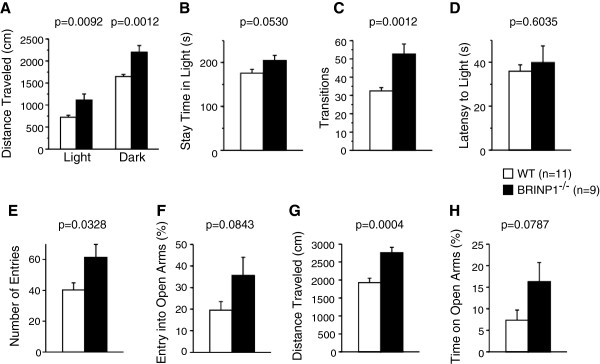
**Anxiety-related behavior of BRINP1-KO mice in light/dark transition and elevated plus maze. (A-D)** Light/dark transition, **(E-H)** Elevated plus maze test. **(A)** Total distance traveled in the light and dark boxes. **(B)** Time the mice stayed in the light box. **(C)** Number of transitions between light and dark boxes. **(D)** Latency time before the first entry into the light box. **(E)** Number of entries into each open and closed arm. **(F)** Percent of entries into the open arms. **(G)** Total distance traveled. **(H)** Percent time spent on the open arms. (wild-type mice, n = 11; BRINP1-KO mice, n = 9) Error bars indicate SEM.

Anxiety-related behavior was also assessed by the light/dark transition test (Figure 4A-D) and the elevated plus maze test (Figure 4E-H) [15]. In the former test, BRINP1-KO mice traveled 1.54- and 1.34-fold longer distances than wild-type mice in both light (WT; 722 ± 45 cm (n = 11), BRINP1-KO; 1113 ± 138 cm (n = 9)) and dark boxes (WT; 1647 ± 48 cm (n = 11), BRINP1-KO; 2200 ± 150 cm (n = 9)), respectively (Figure 4A). In accordance with the longer distances BRINP1-KO mice traveled, the number of transitions between dark and light boxes was 1.62-fold higher in BRINP1-KO mice than in wild-type mice (WT; 33 ± 1.7 (n = 11), BRINP1-KO; 53 ± 5.5 (n = 9)) (Figure 4C). Additionally, BRINP1-KO mice had the tendency to spend longer time in the light box than did wild-type mice (Figure 4B). On the other hand, there was no significant difference in latency of the first entry into the light box from the dark box between each mouse genotype (Figure 4D). These results from light/dark transition test also indicate that BRINP1-KO mice are hyperactive. In the elevated plus maze test, BRINP1-KO mice showed a 1.43-fold longer travel distance than wild-type mice (WT; 1926 ± 126 cm (n = 11), BRINP1-KO; 2760 ± 147 cm (n = 9)) (Figure 4G), a similar extent to the open field test (Figure 3A) and light/dark transition (Figure 4A). In accordance with the increased travel distances, BRINP1-KO mice showed 1.53-fold more frequent entries into the arms (WT; 40 ± 5 (n = 11), BRINP1-KO; 61 ± 8 (n = 9)) (Figure 4E). It was inferred that BRINP1-KO mice showed less anxiety from the fact that BRINP1-KO mice had the tendency to enter 1.82-fold more frequently into the open arms (WT; 19.6 ± 3.9% (n = 11), BRINP1-KO; 35.6 ± 8.5% (n = 9)) (Figure 4F) and spent 2.21-fold longer time in the open arms than did wild-type mice (WT; 7.4 ± 2.4% (n = 11), BRINP1-KO; 16.3 ± 4.5% (n = 9)) (Figure 4H). These results from elevated plus maze test again indicate that the increased locomotor activity in BRINP1-KO mice is accompanied by reduced anxiety.

**Figure 5:**
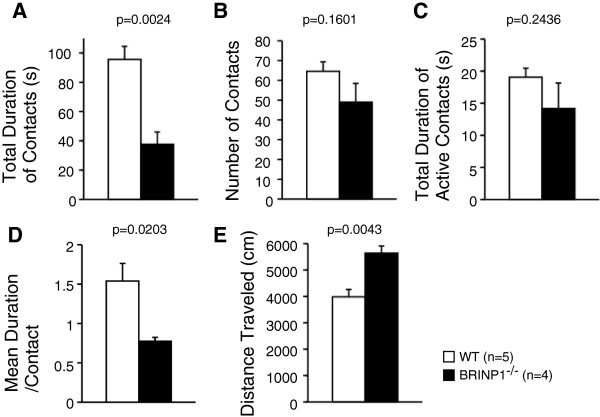
**Social interaction of BRINP1-KO mice in novel environment. (A)** Total duration of contacts. **(B)** Total number of contacts. **(C)** Total duration of active contacts which either of the mice traveled longer than 5 cm. **(D)** Mean duration per contacts. **(E)** Total distance traveled. (wild-type mice, n = 5; BRINP1-KO mice, n = 4) Error bars indicate SEM.

In the social interaction test in a novel environment, BRINP1-KO mice traveled 1.42-fold longer distances than wild-type mice (WT; 3985 ± 282 cm (n = 11), BRINP1-KO; 5641 ± 272 cm (n = 9)) in accordance with other tests mentioned above (Figure 5E). However, both the total duration of contacts (WT; 96 ± 9 s (n = 5); BRINP1-KO; 38 ± 8 s (n = 4)) and the mean duration per contact (WT; 1.5 ± 0.2 (n = 5); BRINP1-KO; 0.8 ± 0.05 (n = 4)) of BRINP1-KO mice were 61% and 50% less than those of wild-type mice, respectively (Figure 5A and 5D). BRINP1-KO mice had the tendency to show a smaller number of total contacts and shorter total duration of active contacts than wild type mice, although differences were not statistically significant (Figure 5B-C). On the other hand, BRINP1-KO mice showed a similar contact pattern in the home cage to wild type mice (see Additional file 1: Figure S1B). Therefore, the marked decrease in the contact durations strongly suggests that BRINP1-KO mice exhibit poor social interaction when encountering an unfamiliar mouse.

**Figure 6:**
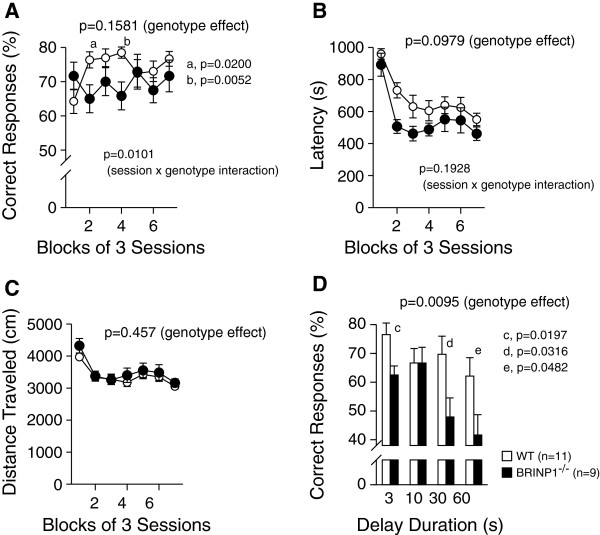
**Working memory of BRINP1-KO mice in T-maze test. (A)** Percentage of correct responses in free-choice run. **(B)** Latency to complete one session. **(C)** Distance traveled within one session. **(D)** Percentage of correct responses when each delay duration was inserted between forced-choice run and free-choice run. (wild-type mice, n = 11; BRINP1-KO mice, n = 9) Error bars indicate SEM.

Working memory of mice was assessed by T-maze alteration test. Percentages of correct responses of BRINP1-KO mice were considerably lower than that of wild-type mice at the certain blocks of 3 sessions during repetitive sessions with short delay period (3 s) (Figure 6A). Although BRINP1-KO mice traveled similar distances compared to wild-type mice during the T-maze test (Figure 6C), they performed the test in shorter time than wild-type mice (Figure 6B). When difficulty of the task was raised by prolonging the delay period (≥30 s), BRINP1-KO mice showed lower performances than wild-type mice (Figure 6D). These results suggest that mild impairment of working memory of BRINP1-KO mice became apparent when the difficulty level was raised.

### Histochemical alterations in BRINP1-KO mice

From the comprehensive behavioral test battery it was revealed that BRINP1-KO mice show several abnormal behaviors: increased locomotor activity, decreased anxiety-like behavior, poor social interaction, and mild deficit of working memory. Since BRINP1-mRNA was highly expressed in the hippocampus and further up-regulated in the dentate gyrus in an activity-dependent manner [4,6], we examined the course of differentiation of dentate granule neurons in BRINP1-KO to elucidate the biological basis of the abnormal behavior.

**Figure 7:**
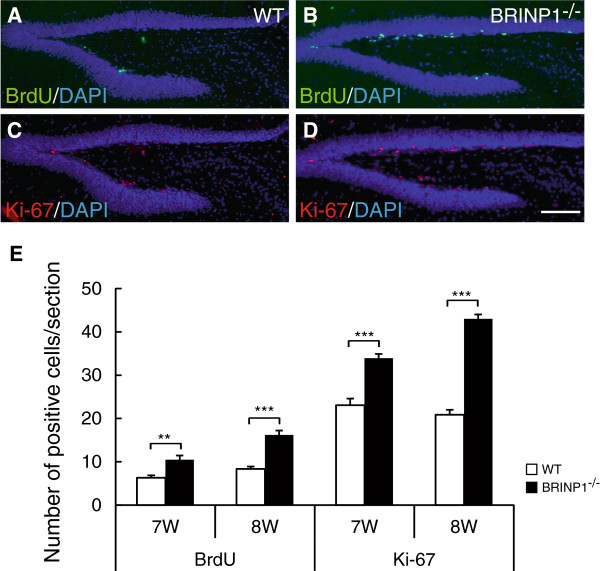
**Adult neurogenesis is increased in BRINP1-KO mice.** Representative images of BrdU positive cells (green) **(A,B)** and Ki-67 positive cells (red) **(C,D)** in dentate gyrus of wild-type mice **(A,C)** and BRINP1-KO mice **(B,D)** at 8 weeks. DAPI in blue. **(E)** Representative graph of the number of BrdU-positive and Ki-67-positive cells in each 7 and 8-week-old mouse dentate gyrus. Data are shown as mean ± SEM. (n = 35 sections per genotype; **: p < 0.01, ***: p < 0.001 Wilcoxon rank-sum test). Scale bar; 100 μm.

**Figure 8:**
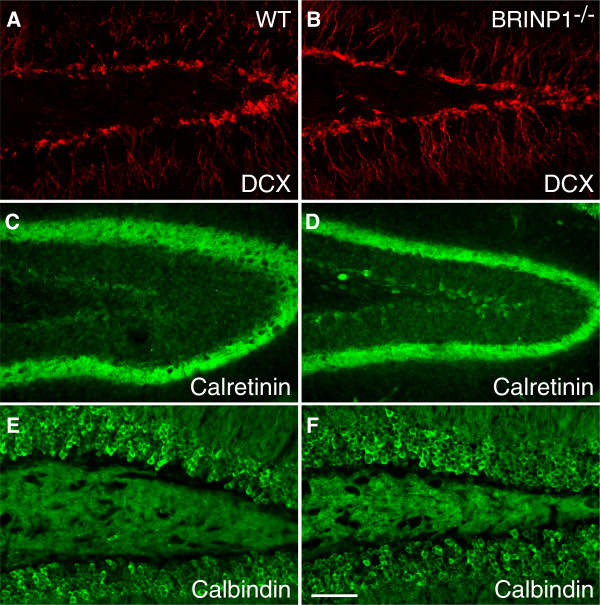
**Neuronal differentiation marker expression in dentate gyrus. (A,B)** Doublecortin, **(C,D)** Calretinin, **(E,F)** Calbindin expression in 7-week-old mouse dentate gyrus. **(A,C,E)** Wild-type mice, **(B,D,F)** BRINP1-KO mice. Scale bar; 50 μm.

To assess the level of adult neurogenesis in SGZ, we examined the number of 5-bromo-2’-deoxyuridine (BrdU)-positive neurons in SGZ after an intraperitoneal injection of BrdU as well as the number of Ki-67-positive neurons in 7 to 10-week-old mice. As a result, the immunoreactivities of both incorporated-BrdU and Ki-67 were noticeably increased in SGZ of 8-week-old BRINP1-KO mice compared to wild-type mice (Figure 7A-D). At 7 and 8-weeks, the average number of BrdU-positive cells per section in BRINP1-KO mice was 1.65- and 1.94-fold greater than those in wild-type mice, respectively (Figure 7E). The number of Ki-67-positive cells in SGZ was also increased in the BRINP1-KO mice in a similar manner to the BrdU-positive cells. On the other hand, in SGZ of 10-week-old mice, there were no obvious differences in the numbers of BrdU- and Ki-67-positive cells between BRINP1-KO mice and wild-type mice (data not shown). These results suggest that BRINP1 function in the hippocampus of normal animals is to suppress neurogenesis in SGZ. Developing dentate granule neurons as well as adult born dentate neurons express several marker proteins depending on their levels of maturation. In the dentate gyrus of 7-week-old BRINP1-KO mice, staining of doublecortin (DCX), a marker for neuroblast and immature neurons [16,17], was stronger than that in wild-type mice (Figure 8A-B). In contrast, expressions of both calretinin, a marker of postmitotic immature neurons and calbindin, a marker of mature neurons in BRINP1-KO mice were similar to those in wild-type mice (Figure 8C-F).

We traced the differentiation of the BrdU-incorporated cells in SGZ with double immunostaining of BrdU and cell type-specific markers; nestin, GFAP, DCX, calretinin, and calbindin. BrdU was administered to both wild-type and BRINP1-KO mice at 11 weeks old and analyzed at different time points from 1 to 5 weeks after BrdU administration. At 2 weeks after administration, 3 times more DCX/BrdU double positive cells were found in BRINP1-KO mice than in wild-type mice. On the other hand, at 4 or 5 weeks after BrdU administration, the numbers of calbindin/BrdU double positive cells were almost the same in both genotypes. These results suggest that in BRINP1-KO mice, the excess newborn neurons in SGZ continue to differentiate to some extent (i.e. to DCX-positive immature neurons) until 2 weeks after their birth, but cannot differentiate further or survive thereafter. Representative images of the DCX/BrdU double positive cells are shown in Additional file 1: Figure S2.

**Figure 9:**
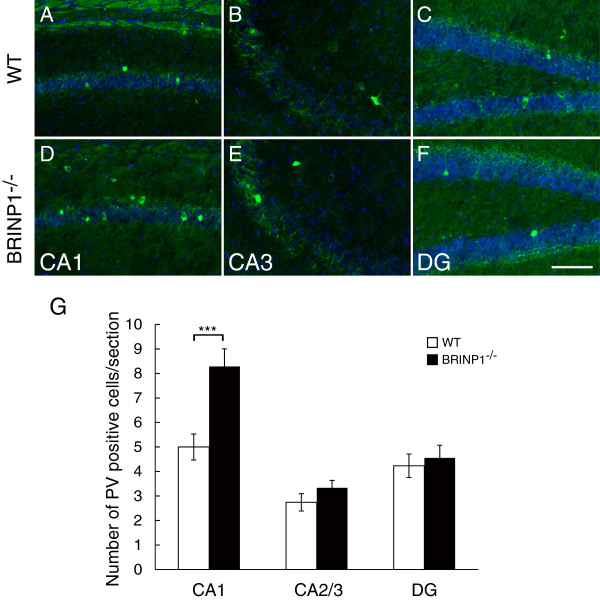
**Parvalbumin expressing interneurons in BRINP1-KO mice.** Representative images of parvalbumin immunoreactivity (green) in hippocampus of wild-type mice **(A-C)** and BRINP1-KO mice **(D-F)**. DAPI in blue. **(G)** The number of parvalbumin expressing cells/section is increased in CA1 subregion of BRINP1-KO mice in hippocampus. Data are shown as mean ± SEM. (n = 26-28 sections from 3 different mice per genotype; ***: p < 0.001 Wilcoxon rank-sum test). Scale bar; 100 μm.

To assess the development of interneuron and glial cells in hippocampus of BRINP1-KO mice, we examined immunostaining of those markers. There was no difference in hippocampal expressions of both glial fibrillary acidic protein (GFAP), a marker for astrocyte, and Iba1, a marker for microglia between BRINP1-KO and wild-type mice (see Additional file 1: Figure S3). The expression level of myelin basic protein (MBP), a marker for oligodendrocyte was also similar in both genotypes (see Additional file 1: Figure S4). The expression level of glutamic acid decarboxylase 67 (GAD67), a common marker for inhibitory interneuron, was also similar in both BRINP1-KO and wild-type mice hippocampus (see Additional file 1: Figure S5). The numbers of large size intensive parvalbumin-expressing neurons in CA2/3 subregion and dentate gyrus are similar in both genotypes, whereas the number of parvalbumin-expressing neurons in CA1 subregion in BRINP1-KO mice was 1.65-fold higher than that in wild-type mice (Figure 9). These results suggest that loss of BRINP1 did not affect the development of glial lineage but increased the population of parvalbumin-expressing interneurons in hippocampal CA1 subregion.

## Discussion

In the present study, from the phenotypic analysis of BRINP1-KO mice, we found that BRINP1 plays multiple roles in the development and maintenance of adult hippocampal circuitry, such as regulation of neurogenesis and neuronal differentiation. Increase of neurogenesis in dentate SGZ and subsequent increase in the population of immature neurons were found in BRINP1-KO mice. The number of parvalbumin expressing interneurons was increased in hippocampal CA1 subregion of BRINP1-KO mice. In addition to these phenotypes, BRINP1-KO mice showed a set of abnormal behaviors: increase in locomotor activity, decreased anxiety-like behavior, poor social interaction, and slight deficit of working memory. These behavioral alterations are similar to symptoms of certain kinds of human psychiatric disorders such as schizophrenia and ADHD. These findings indicate that the BRINP1-KO mouse might serve as a useful animal model in the discovery of drugs to ameliorate symptoms of relevant human psychiatric disorders.

Among the three family members, BRINP1 is most widely expressed in various mouse brain regions including hippocampus, and its expression in adult hippocampus is further up-regulated by the increase of neural activity [6]. In addition to the promoted neurogenesis in hippocampus, BRINP1-KO mice showed a set of abnormal behaviors comparable to the symptoms of human psychiatric disorders. It has been considered that impairments of certain hippocampal circuitry are associated with various neurological diseases such as Alzheimer’s disease, temporal lobe epilepsy, schizophrenia, and mood disorders, even though each disorder displays different regional vulnerability within hippocampal formation [18]. It is highly likely that the abnormal behaviors found in BRINP1-KO mice were evoked by impairments of hippocampal functions.

The increase of neurogenesis in SGZ of adult BRINP1-KO mice suggests that BRINP1 negatively regulates proliferation of adult neural stem cells in normal conditions in accordance with our previous *in vitro* finding. Thus, we reported that over-expression of any of the three BRINPs suppressed the cell-cycle progression at G_1_/S transition in both NIH-3T3 cells [4] and ES-derived neural stem cells (ES-NSCs) [5]. In cultured ES-NSCs, while levels of endogenous three BRINP expressions were very low, they were markedly induced upon differentiation into neuronal cells, but not upon glial differentiation. These results suggest that BRINP1 promotes the entry of newborn cells into quiescent state (G_0_) to establish terminal differentiation during neuronal development and throughout adulthood.

BRINP1-KO mice showed several abnormal behaviors comparable to symptoms of human psychiatric disorders. Previously, several studies on various mutant mice have shown that abnormal behaviors are accompanied by altered neurogenesis [19]. A decrease in adult neurogenesis was reported in mutant mice of schizophrenia-associated genes such as reeler mice [20,21], NPAS3-KO mice [22,23] and DISC1 knockdown mice [24]. Consistent with these findings, a decrease in Ki-67 positive cells was observed also in SGZ of postmortem brain of schizophrenic patients [25]. It has been suggested that the diminished adult neurogenesis is involved also in the reduced cognitive flexibility in major depression, schizophrenia and dementia [26]. In contrast to these observations, we found the increase of adult neurogenesis in SGZ of BRINP1-KO mice. A similar increase of adult neurogenesis was also observed in αCaMKII+/− mice which showed abnormal behavior such as hyperactivity, decreased anxiety-like behavior and working memory deficit [27]. It may seem contradictory that both the increase and decrease in adult neurogenesis contribute to the expression of similar abnormal behaviors. However, the products of these mutated genes would possess distinct physiological functions and influence different aspects of neurogenesis of dentate granule neurons, such as rate of neurogenesis, course of differentiation, synapse formation and cell survival. For example, DISC1 may regulate the rate of development of newborn neurons [28] and modulate GSK3β/β-catenin signaling [24], while NPAS3 regulates reelin expression [29]. It was reported that the neurogenesis in mouse hippocampus declines with age because of the depletion of stem cell pool [30]. It is plausible that increased neurogenesis in mutant mice accelerates the depletion. It has to be clarified whether the decreased neurogenesis and cognitive impairment are the consequences of promoted neurogenesis at earlier stages.

Regardless of the alteration of neurogenesis, abnormal behaviors observed in BRINP1-KO mice are comparable to the symptoms of schizophrenia and ADHD. Deficient mice of both calcineurin [31,32] and Schnurri-2 [33,34] showed increase in locomotor activity, poor social interaction and deficit of working memory. Among the several potential ADHD model mice, both hyperactivity and working memory deficit were observed in deficient mice of dopamine transporter (DAT) [35,36] and gamma aminobutyric acid transporter (GAT) [37]. Mutant mice overexpressing casein kinase 1 (CK1δ) [38] and Grin1 (glutamate receptor, ionotropic, NMDA1) [39] showed increased locomotor activity and abnormal anxiety-like behavior. It has been shown that dysregulation of the complex interplay among monoamines (dopamine, norepinephrine and serotonin) is associated with development of ADHD [40]. Considering the functions of monoamines modulating the fast neurotransmission of glutamate or GABA, abnormal behaviors observed in BRINP1-KO mice might well be caused by alteration of the fast neurotransmission in hippocampus or its modulation by the monoamines. To elucidate the possible link between BRINP1 mutation and abnormal behaviors, genomic mutation in DBCCR1 locus, a human ortholog of BRINP1, should be analyzed in patients of psychiatric disorders.

In the present study of BRINP1-KO mice, we found that excessively generated dentate granule cells can differentiate into DCX-positive immature neurons, but then failed to continue any further differentiation, maturation or survival. The excess production of immature neurons and subsequent elimination may well affect the development or maintenance of neuronal circuitry in hippocampus and eventually lead to the development of abnormal behaviors. It is suggested that BRINP1 is also involved in the particular processes of differentiation, maturation and survival of dentate granule neurons.

In addition to the alteration of DCX expression, BRINP1-KO mice possessed more parvalbumin-expressing interneurons in hippocampal CA1 subregion. It was reported that the number of parvalbumin-expressing inhibitory neurons was decreased in prefrontal cortex in postmortem schizophrenic brain, this deficit of GABAergic input may cause impairment of working memory [41]. Several studies on mutant mice of DISC1, one of the susceptible genes for schizophrenia, showed the reduction of parvalbumin-expressing interneurons in prefrontal cortices [42-44] and CA region of hippocampus [43]. Although the increase of parvalbumin-expressing interneurons in hippocampal CA1 subregion in BRINP1-KO mice is contrary to these observations, DISC1 L100P mutant mice also showed the increase of parvalbumin-positive cells in both CA1 and CA2/3 subregions [45]. The DISC1 L100P mutant mice exhibited schizophrenic-like behaviors including impaired working memory [46]. Thus, both the impairment of working memory and the increase of parvalbumin-expressing neurons in hippocampus were observed in this strain, even though there were controversial results of behavior with DISC1 L100P mutant mice obtained from different genetic background [47]. Parvalbumin-expressing GABAergic neurons innervate cell body of CA1 pyramidal neurons and modulate their glutamatergic output [48]. It was also reported that selective removal of parvalbumin-expressing neurons in CA1 region leads to impairment of spatial working memory [49]. Increased inhibitory innervation on CA1 pyramidal neurons in BRINP1-KO mice may also disturb CA1 output and consequently cause impairment of working memory.

In the present study it was revealed that the loss of BRINP1 causes deregulation of neurogenesis and impairments of neuronal differentiation in adult hippocampal circuitry. In addition, BRINP1-KO mice showed a characteristic set of abnormal behaviors which provides a useful animal model for a group of human psychiatric disorders such as schizophrenia and ADHD. Therefore, confirmation of the causal relationship between alterations of hippocampal neurogenesis and differentiation and abnormal behaviors of BRINP1-KO mice could provide important clues for etiological mechanisms of human psychiatric disorders related to the impairment of hippocampal functions. Future studies focusing on the interactions of BRINP1 and its associated molecules should clarify the molecular mechanisms of the neurogenesis, differentiation and survival of hippocampal neurons.

## Conclusions

In summary, we generated BRINP1-KO mice and analyzed their behavioral and histochemical alterations. BRINP1-KO mice showed abnormal behaviors: hyperactivity, decreased anxiety-like behaviors, poor social interaction, and slight impairment of working memory, all of which resemble symptoms of certain human psychiatric disorders. Absence of BRINP1 causes increase of adult neurogenesis in SGZ and alteration of neuronal differentiation in hippocampus. These alterations found in hippocampal neurons might be responsible for abnormal behaviors in BRINP1-KO mice. The disturbed hippocampal circuitry formed by BRINP1-deficiency could serve as an endophenotype for human psychiatric disorders such as schizophrenia and ADHD.

## Materials and methods

### Generation of BRINP1-KO mice

A targeting vector for homologous recombination was designed to replace exon8 (containing 50% of BRINP1 coding sequence) of mouse *Brinp1* gene with a PGK-Neo cassette from pKJ2 [50] (Figure 1A). Approximately 10 kb genomic DNA region which encompass entire targeting vector was obtained by screening of mouse genomic library and genomic PCR of TT2 mouse embryonic stem (ES) cell [51] (Gibco-BRL) genomic DNA. Diphtheria toxin A fragment (DT-A) from pMCDT-A (A + T/pau) [52] was incorporated in the targeting vector with the purpose of negative selection. The targeting vector was linearized with NotI and electroporated into TT2 ES cells. ES cells were maintained as described previously [53] except that 15% KnockOut Serum Replacement (Gibco by life technologies) was used instead of 20% fetal bovine serum. Transfected cells were selected in the presence of 150 μg/ml Geneticin (Gibco by life technologies) for 8–10 days. G418 resistant clones were screened by genomic PCR using forward primers specific to wild-type allele (BP1 ex8 TV2 D1: 5’-ATGCTGACCTCCTACGAAGT-3’) or specific to the mutant allele (BP1 ex8 C2 neo: 5’-TCTGGATTCATCGACTGTG-3’) in combination with a common reverse primer (BP1 ex8 TV2 A1: 5’-TGTGAATGGCATATAAGACTGATC-3’). The positive ES clones were subjected to Southern blot analysis to confirm the accomplishment of homologous recombination. Eight-cell stage embryos were collected from ICR mice and the zonae pellucidae were removed, and were then aggregated with ES cells with the resulting blastocysts transferred to the uterus of pseudopregnant ICR female mice. Male chimeric offspring were crossed with C57BL/6J female mice to obtain heterozygous mice. Genotypes were determined by genomic PCR using forward primers (BP1 ex8 TV2 D1 and BP1 ex8 C2 neo) in combination with a common reverse primer (BP1 ex8 g34: 5’-CTGAGACCCTTCTTCATGAC-3’). BRINP1 heterozygous mice were backcrossed onto C57BL/6J mice to obtain a congenic strain. The achievement of backcross breeding was assessed by PCR-SSLP (simple sequence length polymorphism) using 43 different microsatellite markers and 97% of marker’s amplicon sizes were identical to those of C57BL/6J mice at 6th backcross generation.

### Southern blot & Northern blot

Forty μg each of genomic DNA extracted from ES cells and mouse tail was digested with BamHI. The resultant fragments were electrophoresed on 0.8% agarose gel and transferred to nitrocellulose membrane. Then, the blot was hybridized with 675 bp ^32^P-labeled DNA probe which is located at a region adjacent to the long arm of *Brinp1* exon8 targeting vector. Radioactive signals were detected with Image Analyzer FLA-5100 (Fujifilm, Tokyo, Japan).

Northern blot analysis was performed according to the method described previously [54]. Total RNA extracted from the adult whole brain was hybridized with ^32^P-labeled cRNA probes for *Brinp1* (675 nucleotides corresponding to mouse *Brinp1* exon8), *Brinp2* (634 nucleotides corresponding to rat *Brinp2* exon8, 96% identical to mouse *Brinp2*) and *Brinp3* (650 nucleotides corresponding to rat *Brinp3* exon2-5, 96% identical to mouse *Brinp3*), respectively. Prior to hybridization, each membrane was stained with methylene blue to confirm quality and amount of RNA.

### Animals

BRINP1-KO mice and wild-type littermates were obtained by breeding the heterozygote mice which were backcrossed onto a C57BL/6J line for at least 6 generations. Mice were maintained under a normal light/dark cycle (12 h light/12 h dark) with food and water *ad libitum*. ICR and C57BL/6J mice were purchased from Japan SLC, Inc. (Shizuoka, Japan). All animal procedures were performed in accordance with the Guidelines for Animals Experimentation prepared by the Animal Care and Use Committees of Matsuyama University, Hokkaido University and Kyoto University.

### Histochemistry

#### Immunohistochemistry

Animals were deeply anesthetized with sodium pentobarbital, and were exsanguinated by intracardiac perfusion with phosphate buffered saline (PBS) followed by fixation with perfusion of 4% paraformaldehyde (PFA). Whole brain was post-fixed in 4% PFA for 1 h at 4°C and cryoprotected. Each sample embedded in O.C.T. mounting medium (Sakura Finetek) was cut into 12-μm-thick sections at around −20°C and stored on slide glasses at −80°C. Sections were incubated at 4°C over night with primary antibodies: rabbit anti-Ki-67 (1:500, Abcam), goat anti-doublecortin (DCX) (1:1,000, Santa Cruz), rabbit anti-calretinin (1:1,500, SWANT) rabbit anti-calbindin D-28 K (1:400, SWANT), and mouse anti-Parvalbumin (1:7,500, SWANT). Then, the sections were incubated at room temperature for 2 h with fluorescence-conjugated secondary antibodies (donkey anti-goat IgG (H + L, 1:1,000, Biotium), goat anti-rabbit IgG (H + L, 1:1,000, Biotium) or goat anti-mouse IgG1 (1:2,000, Biotium). Signals were detected with a fluorescence microscope (AxioImager M1, Zeiss).

#### BrdU staining

DNA synthesizing cells in the subgranular zone (SGZ) of the hippocampus were evaluated by incorporation of 5-bromo-2’-deoxyuridine (BrdU). BrdU labeling reagent (mixture of BrdU and 5-fluoro-2’-deoxyuridine, GE Healthcare) was administered intraperitoneally to mice at 10 ml/kg and the brain was removed 3 h after. Following the postfixation with 4% PFA for 15 min, 16-μm-thick cryosections were rinsed in PBS and were treated with 10 mM citrate buffer pH6.0 for 10 min in a food steamer [55] for the purpose of antigen-retrieval. Incubation with antibodies was performed, as described above, with anti-BrdU antibody (1:1,000, Millipore, clone BU-1) and CF488A goat anti-mouse IgG_2a_ (1:2,000, Biotium) sequentially. In each mouse, 35 serial sagittal sections beginning 0.48 mm from midline were used for analysis.

### Behavior

All behavioral tests were carried out with male mice that were at least 10 weeks old at the start of testing. Statistical analysis was conducted using StatView (SAS Institute, Cary, NC). Data were analyzed by one-way ANOVA, two-way ANOVA, or two-way repeated-measures ANOVA, unless noted otherwise. Values in graphs are expressed as the mean ± SEM**.** The raw data of behavioral tests, which are not described in this paper, are disclosed in the Mouse Phenotype Database (http://www.mouse-phenotype.org/).

#### Motor function tests

The neuromuscular strength of the mice was examined by measurement of grip strength and hanging wire tests. A grip strength meter (O’Hara & Co., Tokyo, Japan) was used to assess forelimb grip strength. The mice were lifted and held by the tail so that their forepaws could grasp a wire grid. The animals were then gently moved backwards by pulling the tail with their frontal plane parallel to the surface of the table until they released the grid. The peak force obtained by the forelimbs of the mouse was recorded in Newtons (N). Each mouse was tested three times, and the greatest value was used for statistical analysis. In the wire-hang test, the mouse was placed on a wire lid and waved gently until the mouse gripped the wires, then the wire lid was turned upside down. Latency to fall from the wire lid was measured.

#### Rotarod test

Motor coordination and balance of the mice were measured by the performance on the rotarod. Mice were placed on the rotating drums (3 cm diameter) of an accelerating rotarod (UGO Basile Accelerating Rotarod, Varese, Italy) and then mice were forced to walk constantly to avoid falling from drums. The speed of the rotarod accelerated from 4 to 40 rpm over 5-min period and the latency to fall from the drum was measured.

#### Hot plate test

The hot plate test was used to evaluate sensitivity of mice to a painful stimulus. The mouse was placed on a hot plate (Columbus Instruments, Columbus, OH) kept at 55.0 ± 0.3°C, and latency to the first hind-paw response, such as shaking a foot or licking a paw, was recorded.

#### Open field test

An open field test was used to evaluate the exploratory activity of mice, and also to assess locomotor activity and anxiety in a novel environment. Each mouse was placed in the center of the automated open field apparatus (40 × 40 × 30 cm; Accuscan Instruments, Columbus, OH). Total distance traveled (in cm), vertical activity (rearing, measured by counting the number of photobeam interruptions), time spent in the center (20 × 20 cm), the beam-break counts for stereotyped behaviors, and number of fecal boli were all recorded. Data were collected for 120 min.

#### Light/dark transition test

A light/dark transition test was used to evaluate anxiety of the mice and was performed as described previously in [56]. The apparatus used for the light/dark transition test consisted of a cage (21× 42 × 25 cm) divided into two equal sections by a partition which included a door (O’Hara & Co., Tokyo, Japan). One chamber was brightly illuminated (390 lx), while the other was kept dark (2 lx). Mice were placed into the dark side and allowed to move freely between the two chambers with the door open for 10 min. The total number of transitions between the chambers, time spent in the each side, and the first latency to enter the light side as well as distance traveled (in cm) were recorded automatically.

#### Elevated plus maze

The elevated plus maze was used to evaluate anxiety-related behavior, revealed by conflict in the mouse between a tendency to explore a novel environment and an aversion to a bright and open area. The test was performed as previously described in [57]. The elevated plus maze (O’Hara & Co., Tokyo, Japan) consisted of two open arms (25 cm × 5 cm) and two closed arms of the same size. The closed arms were enclosed with 15 cm high transparent walls. The arms and central square were made of white plastic plates and were elevated to a height of 55 cm above the floor. To minimize the likelihood of animals falling from the apparatus, 3 mm high plastic ledges were provided for the open arms. Arms of the same type were arranged opposite to each other. Each mouse was placed in the central square of the maze (5 cm × 5 cm), facing one of the closed arms. The level of lighting in the room was adjusted to 100 lx. Mouse behavior was recorded during a 10-min test period. The numbers of entries into, and the time spent in the open and enclosed arms, were recorded. For the analysis of data, we used the following four measures: the percentages of entries into the open arms, the time spent in the open arm (s), the number of total entries, and the total distance traveled (cm). Data acquisition and analysis were performed automatically using Image EP software.

#### Social interaction (novel environment)

Two mice of identical genotypes previously housed in different cages were placed together into a box (40 × 40 × 30 cm) and allowed to explore freely for 10 min. Social behavior was monitored by a CCD camera, which was connected to PC. Analysis was performed automatically using Image SI software. Total duration of contact, the number of contacts, the number of active contacts, mean duration per contact, and total distance traveled were measured. The number of active contacts was defined as follows. Images were captured at 1 frame per second, and distance traveled between two successive frames was calculated for each mouse. If the two mice made physical contact with each other and the distance traveled by either mouse was longer than 5 cm, the behavior was considered as ‘active contact’.

#### T-maze alteration test (working memory)

A forced alteration T-maze was used to evaluate working memory in the mice. Apparatus and experimental procedures of T-maze are described in detail elsewhere [58]. Briefly, a mouse was pre-trained to enter either right or left compartment at the ends of T-shape alley to get a sucrose pellet. A single trial of forced alteration task consisted of a forced-choice run and subsequent free-choice run. In the forced-choice run, the mouse stayed in the start box until the door was opened. At the same time, the entrance door for either the right or left compartment was automatically opened, and mouse entered the opened compartment to get the pellet. After consuming the pellet, the exit door leading to the start box was opened and mouse returned to the start box. In the subsequent free-choice run, after a 3-s delay, both right and left entrance doors were opened, and the mouse chose either left or right compartment. If the mouse entered the compartment opposite to the one which was opened in forced-choice run, it received a pellet (correct response). If the mouse entered the same compartment as in the forced-choice run, it was confined for 10 s with doors closed as a penalty. One session consisted of 10 consecutive trials (Figure 6A-C). After 21 sessions, another session was performed with extended delay times (10, 30, 60 s) to increase the difficulty of the task (Figure 6D).

#### Social interaction test in home cage

Social interaction monitoring in the home cage was conducted as previously described [32]. The apparatus used in the home cage was same as that used in locomotor activity. Two mice of the same genotypes that had been housed separately were placed together in a home cage. Their social behavior was then monitored for 9 days. Images from each cage were captured at a rate of one frame per second. Social interaction was measured by counting the number of particles detected in each frame: two particles indicated that the mice were not in contact with each other; and one particle (i.e., the tracking software could not distinguish two separate bodies) indicated contact between the two mice. We also measured locomotor activity during these experiments by quantifying the number of pixels that changed between each pair of successive frames. Data were averaged over three days (from 4th day to 6th day, Additional file 1: Figure S1). Analysis was performed automatically using Image HA software.

## Abbreviations

ADHD: Attention-deficit/hyperactivity disorder; BRINP: BMP/RA-inducible neural-specific protein; BrdU: 5-bromo-2’-deoxyuridine; DCX: Doublecortin; DG: Dentate gyrus; ES: Embryonic stem; GAD: Glutamic acid decarboxylase; GABA: Gamma aminobutyric acid; GFAP: Glial fibrillary acidic protein; MBP: Myelin basic protein; PBS: Phosphate buffered saline; PFA: Paraformaldehyde; SGZ: Subgranular zone; WT: Wild-type.

## Competing interests

All authors declare that they have no competing interests.

## Authors’ contributions

MK and IM designed the experiments. TN and KM designed and made targeting vector for BRINP1-KO. TN performed ES cell screening and TK made chimeric mice. MK, RO and NM performed immunohistochemistry. TM designed and KT performed the behavioral test battery. MK drafted the manuscript. All authors read and approved the final manuscript.

## Supplementary Material

Additional file 1: Figure S1 Activity level of BRINP1-KO mice in home cage. **(A)** Activity levels of mice in home cage. **(B)** Physical contacts in home cage. Activity levels were higher during night time in both genotypes. BRINP1-KO mice were significantly hyperactive during night time compared to wild-type mice. Data represent averages of one hour activity during 3 days (4^th^ to 6^th^ day). **Figure S2.** Neuronal differentiation of BrdU-incorporated cells in SGZ in BRINP1-KO mice. Representative images of DCX (red) and BrdU (green) immunostaining **(A-B)** 2 weeks after BrdU administration. Representative images of Calbindin (red) and BrdU (green) immunotaining 4 weeks **(C-D)** and 5 weeks **(E-F)** after BrdU administration. Arrows indicate double positive cells. DAPI in blue. Scale bar; 50 μm. **Figure S3.** Glial marker expression in hippocampus of BRINP1-KO mice. Representative images of GFAP **(A-F)** and Iba1 **(G-L)** expressions in 10 weeks wild-type mice and BRINP1-KO mice hippocampus. No obvious difference was observed between the genotypes in terms of GFAP and Iba1 expression in hippocampus. DAPI in blue. Scale bar; 50 μm. **Figure S4.** Oligodendrocyte marker expression in hippocampus of BRINP1-KO mice. Representative images of MBP expression in wild-type mice **(A-C)** and BRINP1-KO mice **(D-F)**. No obvious difference was observed between the genotypes in terms of MBP expression in hippocampus. Scale bar; 100 μm. **Figure S5.** GAD67 expression in hippocampus of BRINP1-KO mice. Representative images of GAD67 expression in wild-type mice **(A)** and BRINP1-KO mice **(B)**. No obvious difference was observed between the genotypes in terms of GAD67 expression in hippocampus. Scale bar; 200 μm. Click here for file
