# Supplementary material for: Absence of BRINP1 in mice causes increase of hippocampal neurogenesis and behavioral alterations relevant to human psychiatric disorders
Source: Mol Brain. 2014 Feb 14;7:12. doi: 10.1186/1756-6606-7-12 (PMC3928644; doi:10.1186/1756-6606-7-12)
Supplement: Additional file 1: Figure S1 — Activity level of BRINP1-KO mice in home cage. (A) Activity levels of mice in home cage. (B) Physical contacts in home cage. Activity levels were higher during night time in both genotypes. BRINP1-KO mice were significantly hyperactive during night time compared to wild-type mice. Data represent averages of one hour activity during 3 days (4th to 6th day). Figure S2. Neuronal differentiation of BrdU-incorporated cells in SGZ in BRINP1-KO mice. Representative images of DCX (red) and BrdU (green) immunostaining (A-B) 2 weeks after BrdU administration. Representative images of Calbindin (red) and BrdU (green) immunotaining 4 weeks (C-D) and 5 weeks (E-F) after BrdU administration. Arrows indicate double positive cells. DAPI in blue. Scale bar; 50 μm. Figure S3. Glial marker expression in hippocampus of BRINP1-KO mice. Representative images of GFAP (A-F) and Iba1 (G-L) expressions in 10 weeks wild-type mice and BRINP1-KO mice hippocampus. No obvious difference was observed between the genotypes in terms of GFAP and Iba1 expression in hippocampus. DAPI in blue. Scale bar; 50 μm. Figure S4. Oligodendrocyte marker expression in hippocampus of BRINP1-KO mice. Representative images of MBP expression in wild-type mice (A-C) and BRINP1-KO mice (D-F). No obvious difference was observed between the genotypes in terms of MBP expression in hippocampus. Scale bar; 100 μm. Figure S5. GAD67 expression in hippocampus of BRINP1-KO mice. Representative images of GAD67 expression in wild-type mice (A) and BRINP1-KO mice (B). No obvious difference was observed between the genotypes in terms of GAD67 expression in hippocampus. Scale bar; 200 μm. [file 1756-6606-7-12-S1.pdf]

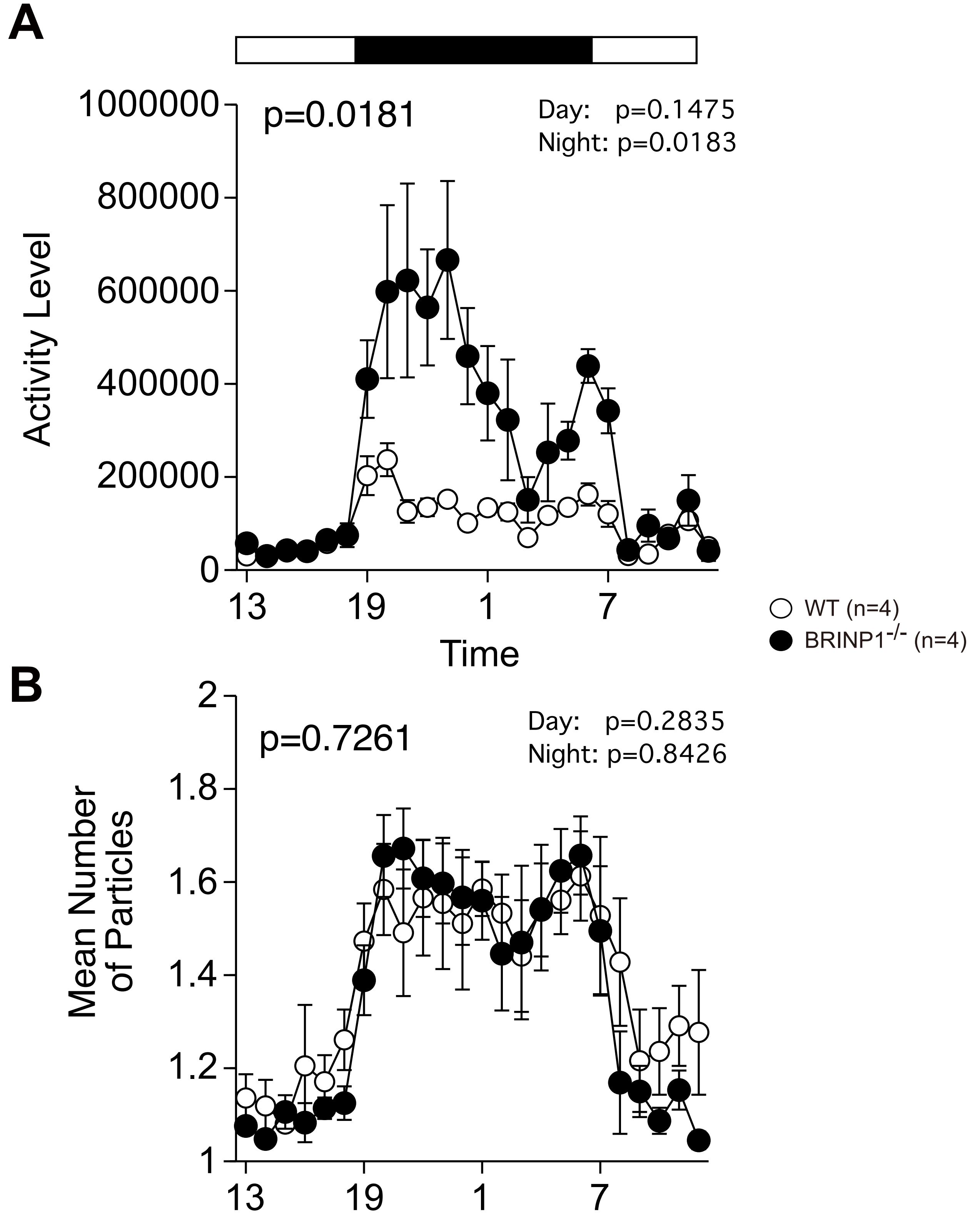

Figure S1 Activity level of BRINP1-KO mice in home cage.

(A) Activity levels of mice in home cage. (B) Physical contacts in home cage.

Activity levels were higher during night time in both genotypes. BRINP1-KO mice were significantly hyperactive during night time compared to wild-type mice. Data represent average of one hour activity during 3 days (4th to 6th d).

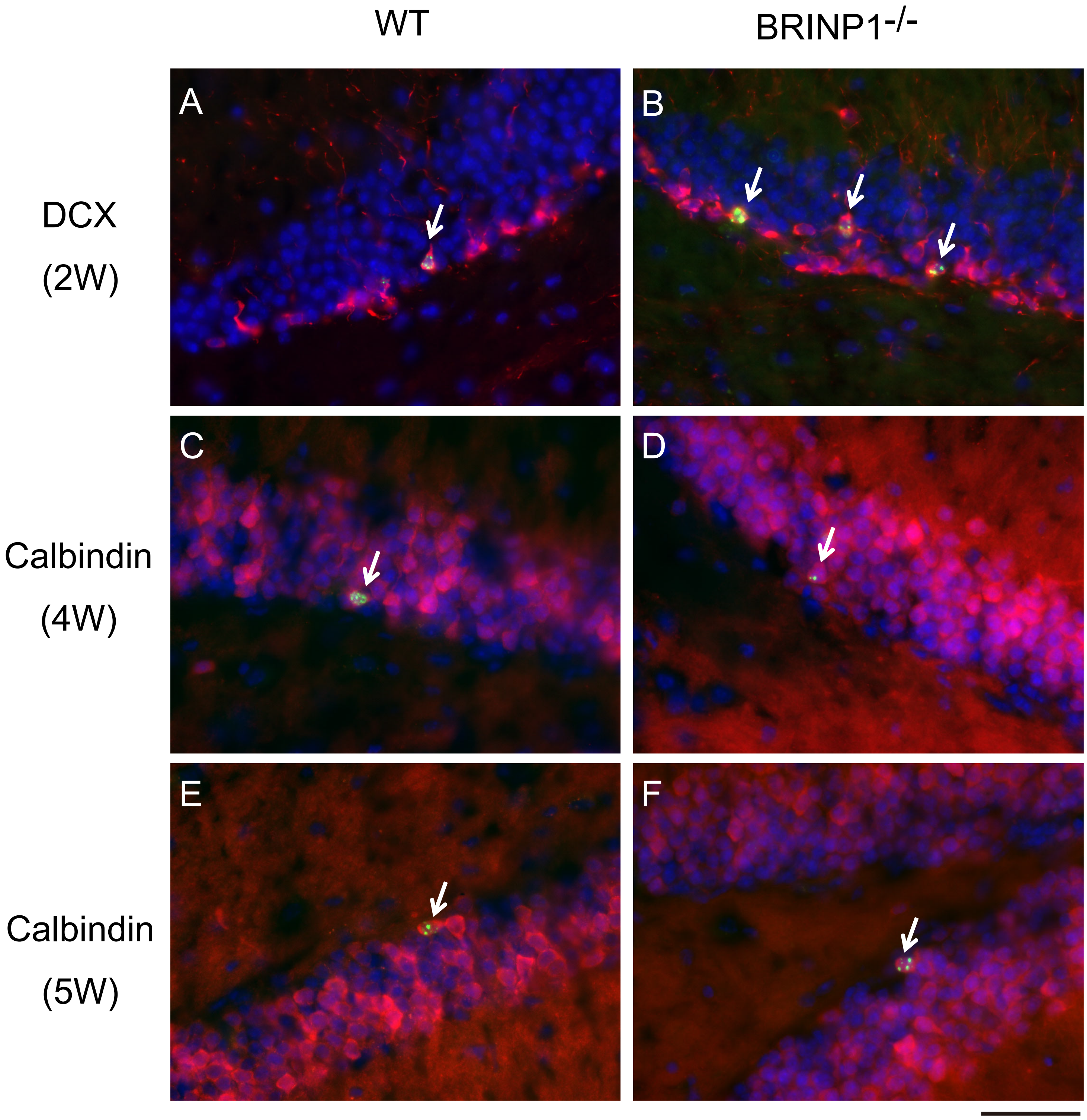

Figure S2 Neuronal differentiaion of BrdU-incorporated cells in SGZ in BRINP1-KO mice. (A,B) Representative images of DCX (red) and BrdU (green) immunostaining 2 weeks after BrdU administration. (C-F) Representative images of Calbindin (red) and BrdU (green) immunotaining 4 weeks (C,D) and 5 weeks (E,F) after BrdU administration. Arrows indicate double positive cells. DAPI in blue. Scale bar; 50 μm

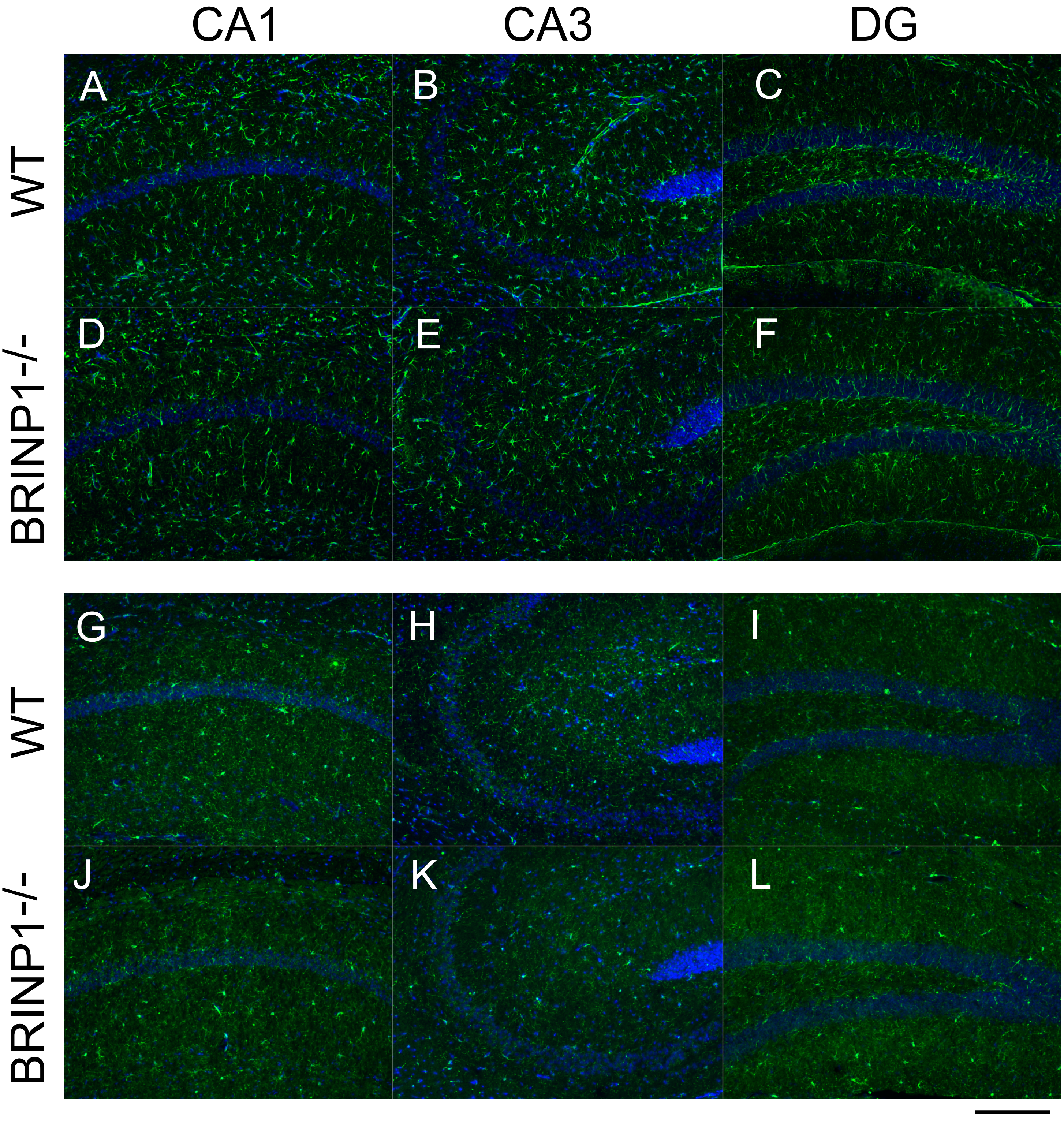

Figure S3 Glial marker expression in hippocampus of BRINP1-KO mice.

(A-F) GFAP (green), (G-L) Iba1 (green) expression in 10 weeks mouse hippocampus. (A-C, G-I) Wild-type mice, (D-F, J-L) BRINP1-KO mice. No obvious difference was observed between the genotypes in terms of both GFAP and Iba1 expression in hippocampus. DAPI in blue. Scale bar; 50 μm

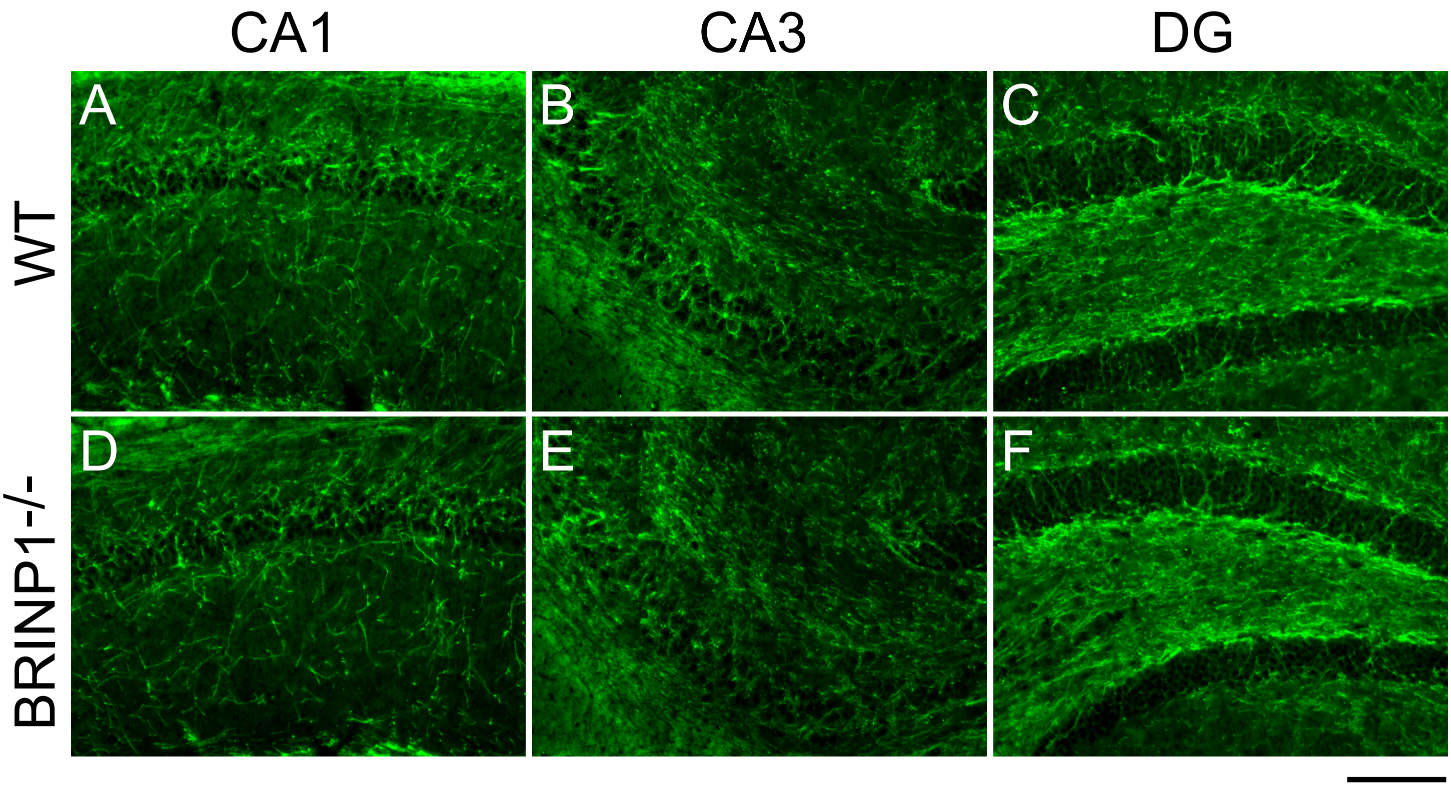

Figure S4 Oligodendrocyte marker expression in hippocampus of BRINP1-KO mice. Representative images of MBP expression in wild-type mice (A-C) and BRINP1-KO mice (D-F). No obvious difference was observed between the genotypes in terms of MBP expression in hippocampus. Scale bar; 100 µm

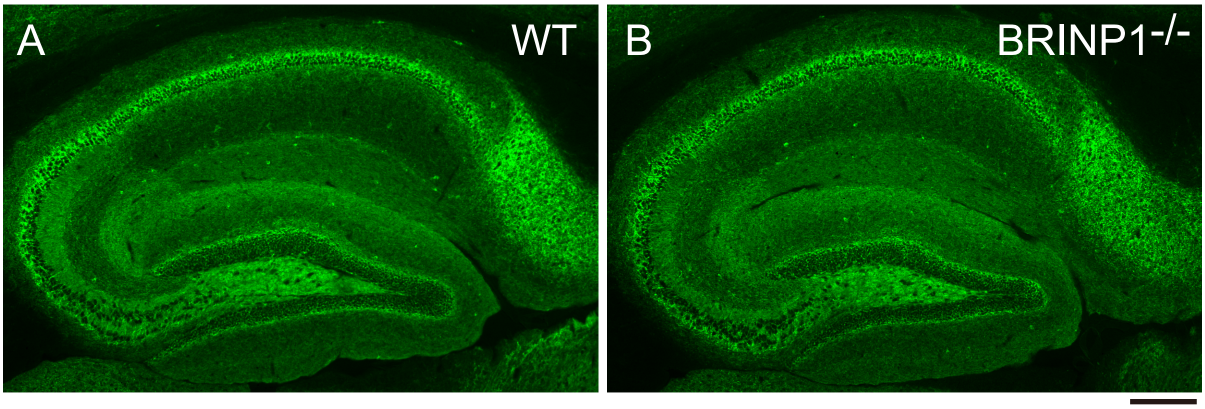

Figure S5 GAD67 expression in hippocampus of BRINP1-KO mice.

Representative images of GAD67 expression in wild-type mice (A) and BRINP1-KO mice (B). No obvious difference was observed between the genotypes in terms of GAD67 expression in hippocampus. Scale bar; 200 μm
